# *In vitro* evaluation of the immunomodulatory and wakame assimilation properties of *Lactiplantibacillus plantarum* strains from swine milk

**DOI:** 10.3389/fmicb.2024.1324999

**Published:** 2024-01-26

**Authors:** Ryusuke Ohgi, Sudeb Saha, Binghui Zhou, Taiga Sakuma, Mitsuki Sakurai, Yuhka Nakano, Fu Namai, Wakako Ikeda-Ohtsubo, Yoshihito Suda, Keita Nishiyama, Julio Villena, Haruki Kitazawa

**Affiliations:** ^1^Food and Feed Immunology Group, Laboratory of Animal Food Function, Graduate School of Agricultural Science, Tohoku University, Sendai, Japan; ^2^Department of Dairy Science, Faculty of Veterinary, Animal and Biomedical Sciences, Sylhet Agricultural University, Sylhet, Bangladesh; ^3^Livestock Immunology Unit, International Education and Research Center for Food Agricultural Immunology (CFAI), Graduate School of Agricultural Science, Tohoku University, Sendai, Japan; ^4^Department of Food Resource Development, School of Food Industrial Sciences, Miyagi University, Sendai, Japan; ^5^Laboratory of Immunobiotechnology, Reference Centre for Lactobacilli (CERELA-CONICET), Tucuman, Argentina

**Keywords:** porcine intestinal epithelial cells, pig milk, *Lactobacillus plantarum*, immunomodulatory potential, wakame

## Abstract

The emergence and spread of antibiotic resistance threat forced to explore alternative strategies for improving the resistance to pathogens in livestock production. Probiotic lactic acid bacteria represent an alternative for this objective. In this study, seven *Lactiplantibacillus plantarum* strains from porcine colostrum and milk were isolated, identified and characterized in terms of their abilities to modulate immunity in porcine intestinal epithelial (PIE) cells. Then, two potential immunoregulatory strains were studied in terms of their ability to utilize and grow in wakame (*Undaria pinnafida*). Isolates were identified by 16S rRNA gene and evaluated by studying their interaction with PIE cells. The expressions of peptidoglycan recognition proteins (PGRPs), nucleotide-binding oligomerization domain (NODs), host defense peptides (pBD), and type I interferons (IFNs) were evaluated by RT-qPCR. The strain 4M_4_417 showed a remarkable capacity to differentially regulate the expression of *PGRP1*, *PGRP3*, *NOD1*, *NOD2*, and *pBD1* in PIE cells. On the other hand, the strain 4M_4_326 was the most efficient to improve the expression of *IFN-α* and *IFN-β* in PIE cells challenged with poly (I:C). Both *L. plantarum* 4M_4_326 and 4M_4_417 were characterized in terms of their ability to utilize wakame. Results demonstrated that both strains efficiently grew in wakame-based broth. Our results suggest that *L. planatrum* 4M_4_326 and 4M_4_417 are interesting candidates to develop immunomodulatory feeds based on wakame utilization. These new immunosynbiotic feeds could help to reduce severity of intestinal infections and improve immune health status in pigs.

## Introduction

Milk is considered as the main source of nutrients for neonates, providing optimal energy and bioactive compounds in their early life (Saha and Ara, 2012; Wang et al., 2022). In addition, it was reported that commensal bacteria from milk are key actors during the early stage of neonatal gut colonization, being major regulators of the host’s immune responses (Stewart et al., 2018). Newborn piglets suckle the sow and rely on the nutrients ingested with milk until weaning (Vodolazska et al., 2023). In general, weaning is performed in piglets around 3–4 weeks when the feeding changes from milk to solid feed. This practice induces a stressful condition to the pig’s life leading to the disruption the gut microbial compositions. This stress can induce alterations of the intestinal morphology and physiological functions and increase the susceptibility to enteric pathogens infections leading to diarrhea (Gresse et al., 2017; Ding et al., 2021). Post weaning diarrhea has been documented as a severe problem after weaning in swine industry globally, and it is one of the leading causes of the mortality observed during this stage. This infectious disease is usually caused by enterotoxigenic *Escherichia coli*, *Salmonella* spp., and rotavirus infection (Cremonesi et al., 2022). To curb post weaning diarrhea there has been a widespread and irrational use of antibiotics as feed additives (Pan et al., 2017) causing the increase of antibiotic resistance in the animals and in the consumers of their products. Thus, the use of antibiotics has been banned in many regions of the world, such as the European Union in 2006 (Chen et al., 2005) or its use has been limited in many countries such as USA, Japan, and China. In order to reduce the use of antimicrobials in livestock, several alternatives are being explored, including the use of immunomodulatory probiotics (immunobiotics) (Villena et al., 2018; Saha et al., 2023).

Recent research has shown that human breast milk is an excellent source of beneficial lactic acid bacteria (LAB) that are vertically transferred to the infant gut (Martín et al., 2007; Qi et al., 2022). Remarkably, some of the LAB strains isolated from breast milk can reduce the severity of infections and inhibit the growth of pathogenic bacteria by competitive exclusions and/or the production of antimicrobial compounds such as bacteriocins or organic acids (Martín et al., 2009). Recent studies have demonstrated that such bacteria could be transferred from the maternal gut to the mammary gland during late pregnancy and lactation through an immunologically based internal route that involves dendritic cells and macrophages: the entero-mammary pathway (Rodríguez, 2014; Selvamani et al., 2021; Qi et al., 2022). The beneficial effects of probiotic LAB in the gut are associated with several mechanisms, including the increase of mucus production, enhancement of epithelial barrier, the competition with harmful microbes for attachment and nutrients in the mucosa, as well as the modulation of the local and systemic immune systems (Elean et al., 2021; Kober et al., 2022). In terms of immunomodulation, it has been demonstrated that the oral administration of probiotic strains can prime the host immune system allowing a faster and more effective response to microbial infections, suggesting that these strains could be used as dietary supplements to enhance innate and adaptive immune system response (Sandes et al., 2017; Abramov et al., 2020).

In general, human breast milk contains approximately 10^3^ to 10^4^ colony forming units/mL of microbes such as Lactobacilli, Lactococci, Enterococci, and *Leuconostoc* spp. (Keddar et al., 2023). Among bacteria present in breast milk, *Lactiplantibacillus plantarum* strains have been identified as potential probiotics (Mollova et al., 2023). *L. plantarum* can survive in the gastrointestinal tract of humans and other mammals including pigs (de Vries et al., 2006; Masumizu et al., 2019; Mollova et al., 2023). In addition, several *L. plantarum* strains have shown to possess beneficial properties for the host, including their ability to beneficially modulate the immune system (Villena et al., 2018). In this regard, we found that different strains of *L. plantarum* such as CRL1506, CRL681, and MPL16 have a remarkable ability to modulate antibacterial and antiviral immune responses (Albarracin et al., 2020; Zhou et al., 2020; Baillo et al., 2022). Considering these facts, we hypothesized that, sow milk contains probiotic *Lactobacillus* strains which may have the capacity to beneficially modulate the immune system of piglets. Few studies have explored the potential probiotic properties of bacterial strains from sow milk (Martín et al., 2009; Gyawali et al., 2015).

In the present work, we aimed to isolate and characterize the immunomodulatory properties of *L. plantarum* from sow milk, particularly focused on their ability to modulate the expression of antimicrobial factors in the context of poly (I:C) challenge in porcine intestinal epithelial (PIE) cells. Considering that research has demonstrated that some prebiotic seaweed can enhance the beneficial effects of probiotic lactobacilli (Makkar et al., 2016; Masumizu et al., 2019), we also evaluated the ability of the strains to grow in wakame (*Undaria pinnafida*), with the aim of developing an immunosynbiotic feed to improve immune health of pigs in the future.

## Materials and methods

### Animals and sow milk sample collection

All pregnant sows (Landrace × Yorkshire × Duroc) were kept at Tsubonuma Farm, Faculty of Food Industry, Miyagi University, Japan. Animals were reared according to the guidelines of the Japanese Association of Laboratory Animal Science (JALAS) for care and use of animals in research. The protocol was approved by the laboratory health and safety committee of Miyagi University (Japan) with an approved protocol no. 2016–2023. The milk sample was collected from healthy pig. Corn-soybean based diet fed to the animals.

Milk samples were collected in a sterile tube. During milk sampling, sterile gloves were worn, nipple and the surrounding area were soaked with 75% ethyl alcohol to avoid the contamination by skin bacteria. The milk samples were immediately refrigerated at 4°C and transferred to the laboratory of Animal Food Function, Tohoku University. After transportation, milk samples were dispensed into serum tubes, mixed with equal amount of 60% (v/v) glycerol (final concentration was 30% glycerol sample stock) and stored at −80°C for the cultivation of bacteria.

### Milk microbiome analysis

For DNeasy Blood & Tissue Kit (Qiagen, United States), 0.2 mL milk sample were centrifuged at 6000 × g for 3 min, total DNA was isolated from its pellet using according to the manufacturer’s instructions for gram positive bacteria and then sent for Bioengineering Lab. Co., Ltd. (Kanagawa, Japan). Total DNA was subjected to 16S rRNA analysis, and read counts were calculated. DNA was amplified using the 2-step tailed PCR to target the V3–V4 regions of bacterial 16S rRNA. 1st PCR was performed with the 1st-341f_MIX (5′-ACACTCTTTCCCTACACGACGCTCTTCCGATCT-NNNNN-CCTACGGGNGGCWGCAG-3′) and the 1st-805r_MIX (5′-GTGACTGGAGTTCAGACGTGTGCTCTTCCGATCT-NNNNN-GACTACHVGGGTATCTAATCC-3′) primers. Subsequently, 2nd PCR was performed with the 2ndF (5′-AATGATACGGCGACCACCGAGATCTACAC-Index2-ACACTCTTTCCCTACACGACGC-3′) and the 2ndR (5′-CAAGCAGAAGACGGCATACGAGAT-Index1-GTGACTGGAGTTCAGACGTGTG-primers). A 16S rRNA metagenomic sequencing library was prepared according to manufacturer’s instructions (Illumina, San Diego, CA, United States). The PCR products were pooled to construct the sequencing library and the quality of the library was confirmed using Fragment Analyzer and dsDNA915 Reagent Kit (Advanced Analytical Technologies). Sequencing was performed using the MiSeq Reagent Kit v3 (Illumina, United States) under the condition of 2 × 300 bp. The paired raw fastq data were merged and quality filtered. The FASTQ data from four breasts underwent analysis within the Quantitative Insights into Microbial Ecology version 2 (Qiime2) platform.^1^ Initially, the dada2 plugin in Qiime2 was employed to filter and denoise the raw FASTQ data. The passed forward and reverse sequences were then merged. Then the chimera sequences were eliminated from the merged sequences to construct the amplicon sequence variant (ASV) table. The ASV tables from four breasts were combined to create one ASV table. Finally, taxonomy analysis was performed by classifying each ASV using the SILVA 138 database (Quast et al., 2012).

### Isolation of *Lactobacillus* from sow milk

To isolate *Lactobacillus* strains, primarily, collected milk samples were thawed and 50 μL of samples were 10-fold serially diluted with sterile phosphate buffered saline (PBS) solution. Later, an aliquot (50 μL) of mixtures was plated onto de Man, Rogosa and Sharpe (MRS, Becton Dickinson Company, United States) and incubated at 37^o^C for 48 h. Single colonies were collected, and pre-cultured onto MRS broth for up to 24 h and stored at −80°C with equal 60% glycerol solution for further experiments. The morphology of isolates was examined by Gram staining, and Gram-positive bacteria were used for 16S rRNA gene sequencing.

### Identification of *Lactobacillus* strains by using 16 s rRNA sequencing

Genomic DNA was extracted from selected bacterial isolates using DNeasy Blood & Tissue Kit (Qiagen, United States) by following the manufacturer’s protocol for gram positive bacteria. The DNA was measured with a Nanodrop ND-2000 spectrophotometer (NanoDrop Technologies Wilimington, DE). 16S rDNA fragment was amplified by PCR using the bacterial universal primers 27f (5´-AGAGTTTGATCCTGGCTCAG-3′) and 1492r (5´-GGTTACCTTGTTACGACTT-3′) (Ref). After amplification, PCR products were purified by gel electrophoresis and the purified PCR products were sequenced by BigDye Terminator v1.1 Cycle Sequencing Kit (Thermo, Co., Ltd., Foster City, CA, United States). The obtained sequences were examined by basic Local Alignment Search Tool (BLAST, https://blast.ncbi.nlm.nih.gov/Blast.cgi) through the alignment of 16 s rRNA gene sequences of isolates with the 16 s rRNA gene sequences of known bacteria in GeneBank database. More than 95% of sequence with a type of strain were used to identify the isolates.

### *In vitro* evaluation of immunomodulatory properties of *L. plantarum* isolates

The porcine intestinal epithelial cell lines (PIE) were used as an *in vitro* cellular model. PIE cells were initially derived from the intestinal epithelium of neonatal unsuckled piglets, previously developed by our research group (Moue et al., 2008), and was maintained in Dulbecco’s Modified Eagle Medium (DMEM) supplemented with fetal calf serum (10%), penicillin (100 U/mL), and streptomycin (100 μg/mL). The PIE cells cultures were grown in a flask. 75 cm^2^ flask at 37°C with 5% CO_2_. The cultures were passaged routinely to reach the confluent 80%–90% and used for experiments between 25th and 35th passages. PIE cells were counted and later seeded at 3.0 × 10^4^ cells in Type I collagen coated 12-well plates (SUMILON, Tokyo, Japan) and incubate at 37°C with 5% CO_2_ for 3 days. On the day 3, *L. plantarum* isolates 5 × 10^7^ cells/well (2.5 × 10^8^ cells/mL) were added and stimulation was continued for 48 h at 37°C with 5% CO_2_.

For TLR3 activation experiments, PIE cells were seeded at 3.0 × 10^4^ cells per well in 12-well type I collagen-coated plates and cultured for 3 days. After changing medium, lactobacilli (2.5 × 10^8^ cells/mL) were added, and 48 h later, each well was washed vigorously with medium at least three times to eliminate all stimulants. Then, cells were stimulated with 5 μL of poly (I:C) (100 ng/mL) (Sigma-Aldrich, St. Louis, MI, United States) for 12 h.

After the treatment with lactobacilli (basal expression) and 12 h after poly (I:C) stimulation, PIE cells were washed twice PBS, treated with 500 μL of TRIzol reagent (Invitrogen, CA, United States) per well, and transferred to a 1.5 mL microtube for RNA extraction according to the manufacturer’s protocol.

### Quantitative real-time PCR analysis

Total RNA was extracted from treated and control PIE cells using TRIzol reagent (Invitrogen, Carlsbad, CA, United States) and reverse transcribed into cDNA using the PrimeScript^™^ RT reagent Kit with gDNA Eraser (Takara Bio Inc., Shiga, Japan) by following the manufacturer’s protocol. The synthesized cDNA was then used for quantitative PCR analysis using Platinum SYBR Green qPCR Super Mix UDG with ROX (Invitrogen, Carlsbad, CA, United States) on a 7,300 real-time PCR system (Applied Biosystems, Warrington, United Kingdom). In brief, cDNA 2.5 μL, Syber MIX 7.5 μL (reverse primer 1.25 μL (1 pM), Platinum SYBR Green qPCR SuperMIX-UDG with POX 5 μL) into each well of the dedicated 96 well plates. Primers used in this study are described in Supplementary Table S1. Amplification was carried out at the following conditions: 50°C for 2 min and 95°C for 5 min, followed by 40 cycles consisting of 95°C for 15 s, 60°C for 30 s, and 72°C for 30 s. β-actin was used as an internal standard to normalize cDNA levels for differences in total cDNA levels in the samples. The samples were run thrice for each experimental condition and the average values were used to statistical analysis.

### Bacterial growth assay

Bacterial growth assay was performed according to our previous publication (Masumizu et al., 2019). In brief, 0.1% (w/v) of wakame powder was suspended in water and the suspension was autoclaved at 121°C for 15 min. The wakame solution adjusted to pH 5 with 1 mol/L HCl and incubated with the two enzymes: 0.25% (w/v) cellulase and 0.25% (w/v) hemicellulose (Mitsubishi-Chemical Foods Corporation, Tokyo, Japan) at 50°C for 24 h. The hydrolyzed wakame was again autoclaved at 121°C for 15 min, and the supernatant was separated by centrifugation at 6,000 rpm for 20 min. The supernatant containing the wakame extract was further supplemented with 0.6% (w/v) Trypticase soy broth and 0.5% (w/v) NaCl. The pH was adjusted to 6.8 and the broth was autoclaved at 121°C for 15 min. This wakame broth obtained at the end of these procedures was considered as ready for use.

The lactobacilli strains used for the study of wakame assimilation were first grown in MRS agar at 37°C for 24 h. A single colony was picked for the pre-culture. After growth, 2% (v/v) of bacteria culture broth was added 5 mL of wakame broth or non-sugar MRS and incubated at 37°C for 48 h. Optical density was monitored for 48 h (measurement interval: 30 min, penetration rate: 60 rpm, total operation time: 48 h) using spectrometer system (TVS026CA, Advantec, Tokyo, Japan).

### Thin layer chromatography

Wakame solution with or without enzyme treatment, the supernatant of the wakame broth fermented with *L. plantarum* strains, and 1% (w/v) of standard saccharides solutions (glucose, galactose, fructose, lactose, mannose, raffinose, alginate, and cellobiose) were dropped on TLC Silica gel 60 (Merck kGaA, Darmstadt, Germany). Butyl alcohol, isopropyl alcohol, and water (3:12:4) mixture was used as the solvent. After 2 times developing, 5% (v/v) sulfuric acid in methanol was sprayed on the plate and it was heated at 150°C for 10 min until spot visualizing.

### Statistical analysis

The statistical analyses were performed by using Bell Curve for Excel (Social Survey Research Information Co., Ltd., Tokyo, Japan). Data were presented as the mean ± SD. Differences between the control were assessed using one-way ANOVA followed by the independent two-tailed *t*-test. Differences were considered as significant when *p* < 0.05.

## Results and discussion

### Isolation of LAB from sow milk

**Figure 1:**
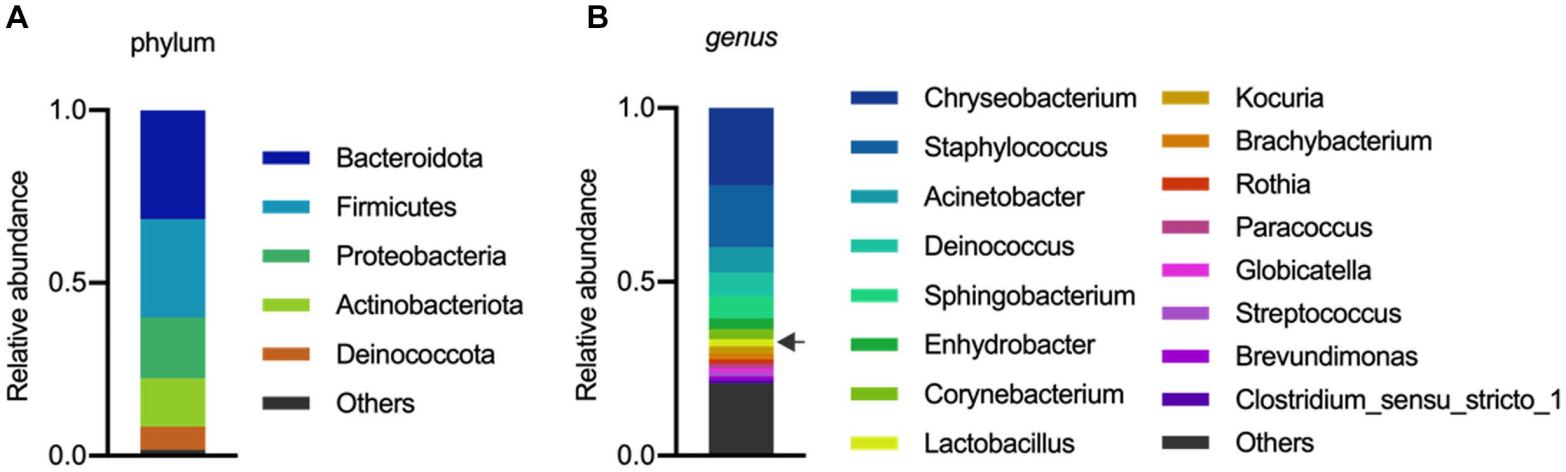
Microbial composition of porcine colostrum (day0) at Phylum level **(A)** and Genus level **(B)**. Microbiome data from four breasts were combined in a single bar plot. The arrow indicates *Lactobacillus*.

The analysis of the microbial composition of porcine colostrum revealed the presence of a complex bacterial community (Figure 1A). *Chryseobacterium* and *Staphylococcus* were abundant genera, with *Lactobacillus* comprising approximately 2% of the colostrum microbiome (Figure 1B). Our results are consistent with previous studies that have identified porcine milk as a rich source of *Lactobacillus* (Martín et al., 2009; Wang et al., 2022). In a study by Chen et al. (2018) demonstrated that the microbial composition and diversity of the porcine colostrum changed significantly but was relatively stable in transitional and mature milk. The work described that the genera *Corynebacterium* and *Streptococcus* were significantly higher in colostrum while the bacterial groups *Lactobacillus*, *Ruminococcaceae*, unclassified *Lachnospiraceae*, and unclassified *Clostridiales* were more abundant in milk. In addition, *L. reuteri* was the most dominant species in milk (Chen et al., 2018). However, it should be noted that the milk microbiota of sows has not been systematically investigated, therefore there is not enough information to compare similarities and differences between studies and draw robust conclusions.

Next, we aimed to isolate LAB strains for samples using MRS plates. Most of the isolated strains belonged to the species *W. thailandensis*, *W. cibaria*, *W. paramesenteroides*, and *L. lactis* (Supplementary Table S2). The identification by 16S rDNA PCR of isolated colonies revealed that among lactobacilli all the strains belonged to the species *L. plantarum* (Supplementary Table S2). Thus, our experimental procedures did not allow the isolation of other lactobacilli species present in the porcine milk. Similarly, the study of Wang et al. (2022) showed that from a total of 1,240 isolates from porcine milk, 922 belonged to the group of *Lactobacillales*, which was dominated by *L. lactis*. In addition, *L. reuteri*, *L. salivarius*, *L. plantarum*, *L. paraplantarum*, *L. brevis*, and *W. paramesenteroides* were the most abundant species isolated from sow milk (Martín et al., 2009). Considering that we were interested in *L. plantarum* strains because of their potential higher capacity to grow in wakame (Masumizu et al., 2019), we selected seven strains belonging to this species of lactobacilli (4cs321, 4cs331, 4M_4_326, 4M_4_338, 4M_4_346, 4M_4_347, and 4M_4_417) for the evaluation of the immunomodulatory properties in PIE cells.

### Immunoregulatory properties of *L. plantarum* isolates

**Figure 2:**
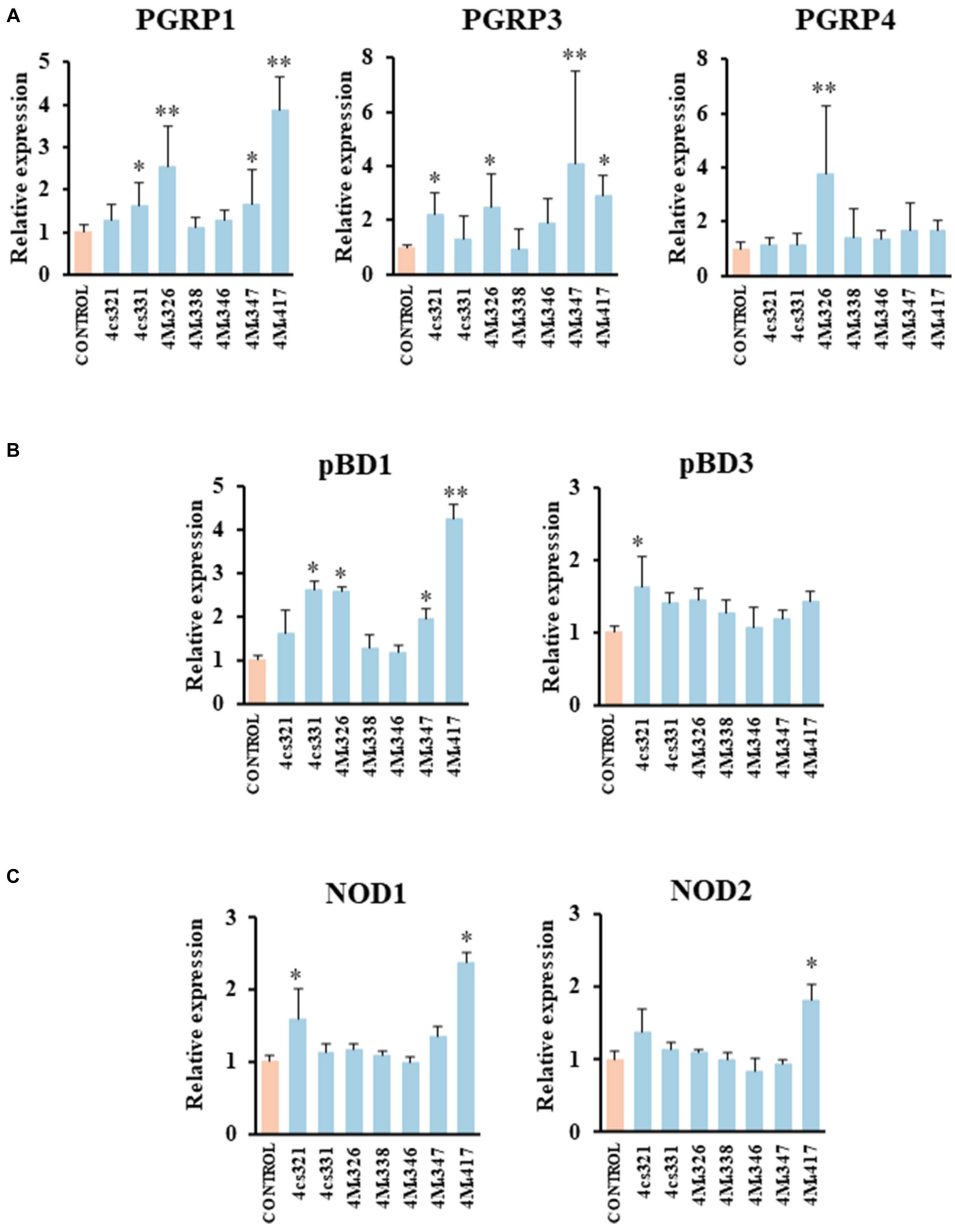
Effect of porcine lactobacilli on the expression of peptidoglycan recognition proteins (PGRP) **(A)** PGRP1, PGRP3, PGRP4, **(B)** host defense peptides pBD1, pBD3, and nucleotide-binding oligomerization domain (NOD), **(C)** NOD1 and NOD2 in PIE cells. The cells were stimulated with different *Lactiplantibacillus plantarum* strains (4cs321, 4cs331, 4M_4_326, 4M_4_338, 4M_4_346, 4M_4_347, and 4M_4_417) and the expression of immune factors was evaluated. PIE cells without lactobacilli stimulation were used as control. Values are presented as mean ± SD of three independent experiments (*n* = 3 per experiment). *p < 0.05 and **p < 0.01.

We demonstrated previously that PIE cells are a useful *in vitro* tool to evaluate the ability of beneficial bacteria to modulate the innate immune responses, which has a high correlation with posterior *in vivo* studies (Shimazu et al., 2012; Suda et al., 2014; Albarracin et al., 2020). Then, in a first set of experiments we assessed whether the pre-stimulation of PIE cells with lactobacilli differentially modulate the expression of immune genes in PIE cells stimulated with the isolates of *L. plantarum* (Figure 2). A clear strain-dependent effect was observed when the expressions of *PGRPs*, *NODs*, and *pBDs* genes were analyzed, which is in line with our previous reports evaluating LAB strains of different origins (Villena et al., 2016; Masumizu et al., 2019; Mizuno et al., 2020; Baillo et al., 2022).

*L. plantarum* 4M_4_326 was the only strain able to increase *PGRP4* expression. This strain also upregulated *PGRP1* and *PGRP3*. *L. plantarum* 4cs331 increased *PGRP1* while the 4M_4_321 strain upregulated *PGRP3* (Figure 2A). The most remarkable effect was found for *L. plantarum* 4M_4_417 that enhanced the expression of *PGRP1*, and *PGRP3* in PIE cells. PGRPs possess antibacterial and immunomodulatory properties that allow them to actively participate in the interaction of host’s cells with the intestinal microbiota. It was reported that mice lacking any of the four genes coding for PGRPs are more susceptible to dextran sulfate sodium (DSS)-induced colitis (Saha et al., 2010). Furthermore, stools from mice deficient in PGRPs when transferred to wild type germ-free mice can increase the severity of colitis when compared to the stools from normal mice (Jing et al., 2014). Although significant advances have been made in the biology of PGRPs in human and mice, their functions in pigs have been less explored. Our group demonstrated that the four PGRPs are expressed in PIE cells and that their expression can be modulated by probiotic microorganisms (Iida et al., 2019). Our data provided evidence of the potential capacity of the modulation of PGRPs by beneficial microbes to enhance resistance and reduce inflammatory damage severity during intestinal infections.

The results showed that *L. plantarum* 4cs321 significantly upregulated the expression of pBD3 while the 4cs331 strain increased *pBD1* in PIE cells stimulated with the isolates of *L. plantarum* when compared to controls (Figure 2B). The expression of *pBD1* was also enhanced by the strains 4M_4_326 and 4M_4_347. *L. plantarum* *4M4417* significantly increased the expression of *pBD1* (Figure 2B). The secretion of host defence peptides by the intestinal epithelium is of importance since these factors can exert both antimicrobial and immunomodulatory activities, providing an early response to bacterial infections in the gut (Veldhuizen et al., 2008). Interestingly, the treatment of weaned piglets with synthetic host defence peptides was shown to improve their growth performance, nutrient digestion, and intestinal health (Yoon et al., 2013). An alternative to the administration of synthetic peptides is the induction of their expression using beneficial microorganisms. Some studies have shown the ability of *L. plantarum* strains to differentially modulate the expression of host defence peptides in porcine intestinal epithelial cells. The probiotic strain *L. plantarum* ZLP001 was shown to upregulate the expression of *pBD2* (a β-defensin) and *PG1-5* (a cathelicidin) in IPEC-J2 cells (Wang et al., 2018) in a TLR2/MAPK/AP-1 signaling dependent pathway (Wang et al., 2019). This effect has been described as one of the mechanisms involved in the augmented growth performance in post-weaning piglets upon the treatment with the ZLP001 strain (Wang et al., 2012). Similar studies have demonstrated the ability of lactobacilli to modulate host defence peptides in porcine intestinal epithelial cells including *L. reuteri* I5007 (Liu et al., 2017), *L. salivarius* B1 (Zhang et al., 2011), and *L. plantarum* Lac16 (Zhou et al., 2021).

We also investigated the effect of *L. plantarum* isolates on the expression of *NOD1* and *NOD2* in PIE cells (Figure 2C). The expression level of *NOD1* was increased in PIE cells treated with the 4cs331 strain while *L. plantarum* 4M_4_417 was the only one with the capacity to improve the expression of both *NOD1* and *NOD2*. The modulation of the expression of *NOD1* and *NOD2* in the intestinal epithelium has been connected to the ability of the intestinal microbiota and probiotics to beneficially modulate the inflammatory response. Studies in specific pathogen-free *Nod1*^−/−^ and *Nod2*^−/−^ mice showed that both receptors are necessary for the intestinal microbiota or probiotics to reduce the severity of colitis (Natividad et al., 2012).

**Figure 3:**
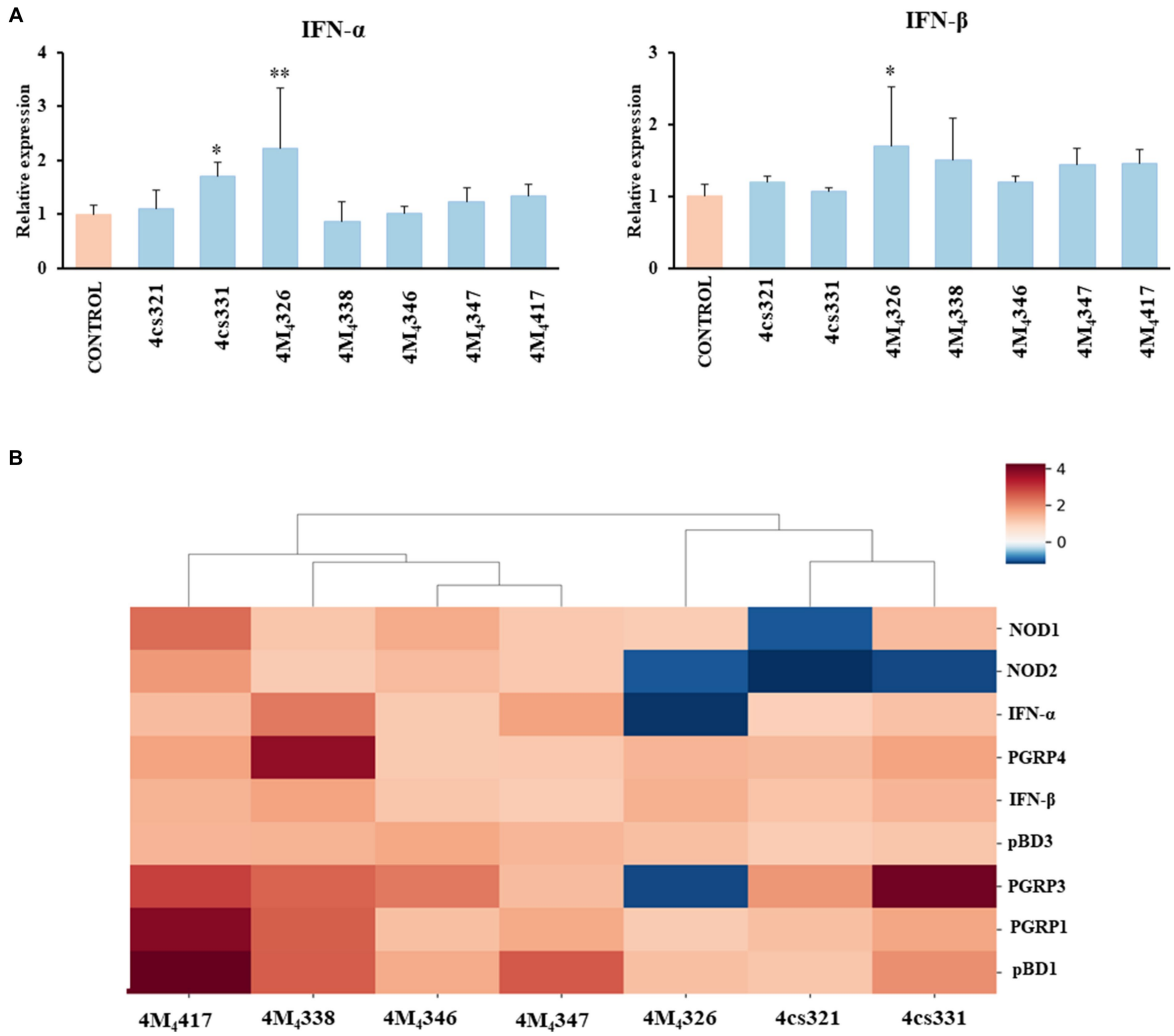
Effect of porcine lactobacilli on the expression of type I interferons (IFNs) **(A)** IFN-α and IFN-β in PIE cells challenged with poly (I:C). PIE cells were stimulated with different *Lactiplantibacillus plantarum* strains (4cs321, 4cs331, 4M_4_326, 4M_4_338, 4M_4_346, 4M_4_347, and 4M_4_417) and then challenged with poly (I:C). PIE cells without lactobacilli treatment and challenged with poly (I:C) were used as controls. Values are presented as mean ± SD of three independent experiments (*n* = 3 per experiment). *p < 0.05 and **p < 0.01. Heat map analysis **(B)** of the differentially regulated genes in PIE cells treated with *L. plantarum* strains and challenged with poly (I:C).

On the other hand, we evaluated the ability of the seven *L. plantarum* strains from porcine milk to modulate TLR3-mediated immune responses in PIE cells. For this purpose, cells were stimulated with lactobacilli, challenged with the TLR3 agonist poly (I:C), and then the expression of *IFN-α* and *IFN-β* was evaluated (Figure 3A). Like the experiments with *L. plantarum* stimulation (Figure 2), a clear strain-dependent effect was observed when the expressions of type I IFNs genes were analyzed, which is in line with our previous reports evaluating LAB strains of different origins (Albarracin et al., 2020; Zhou et al., 2020). Most of the strains had no effect on the expression of *IFN-α* and *IFN-β* in PIE cells stimulated with poly (I:C) (Figure 3A). The strain 4cs331 increased the expression of *IFN-α* while the most remarkable effect was detected for *L. plantarum* 4M_4_326 that significantly enhanced both *IFN-α* and *IFN-β*. We previously reported that *L. plantarum* MPL16 and *L. plantarum* CRL1506 had the capacity to enhance the expression of type I IFNs and antiviral factors in poly (I:C)-challenged PIE cells, an effect that is not sheared by strains of the same species (Albarracin et al., 2017, 2020). Of note, both CRL1506 and MPL16 strains efficiently regulate the production of *IFN-α* and *IFN-β* in the gut of mice challenged with poly (I:C), which indicates that results obtained in PIE cells could be extrapolated to *in vivo* situations (Albarracin et al., 2020).

Taken together, our results show that some of the *L. plantarum* strains isolated from porcine milk have an excellent potential to be used in the formulation of immunomodulatory feeds and to be tested *in vivo* in pigs. To establish the best strains for further studies, we jointly analyzed gene expression changes in the two experiments (*L. plantarum* strains and poly (I:C) stimulation) using a heat map (Figure 3B). All the strains were clearly differentiated from each other, observing the most notorious effect for *L. plantarum* 4M_4_417. This strain was selected for the following studies. In addition, the strain 4M_4_326 was selected because was the only one with a noticeable effect on the modulation of antiviral immunity.

### Wakame utilization ability of *L. plantarum* strains

**Figure 4:**
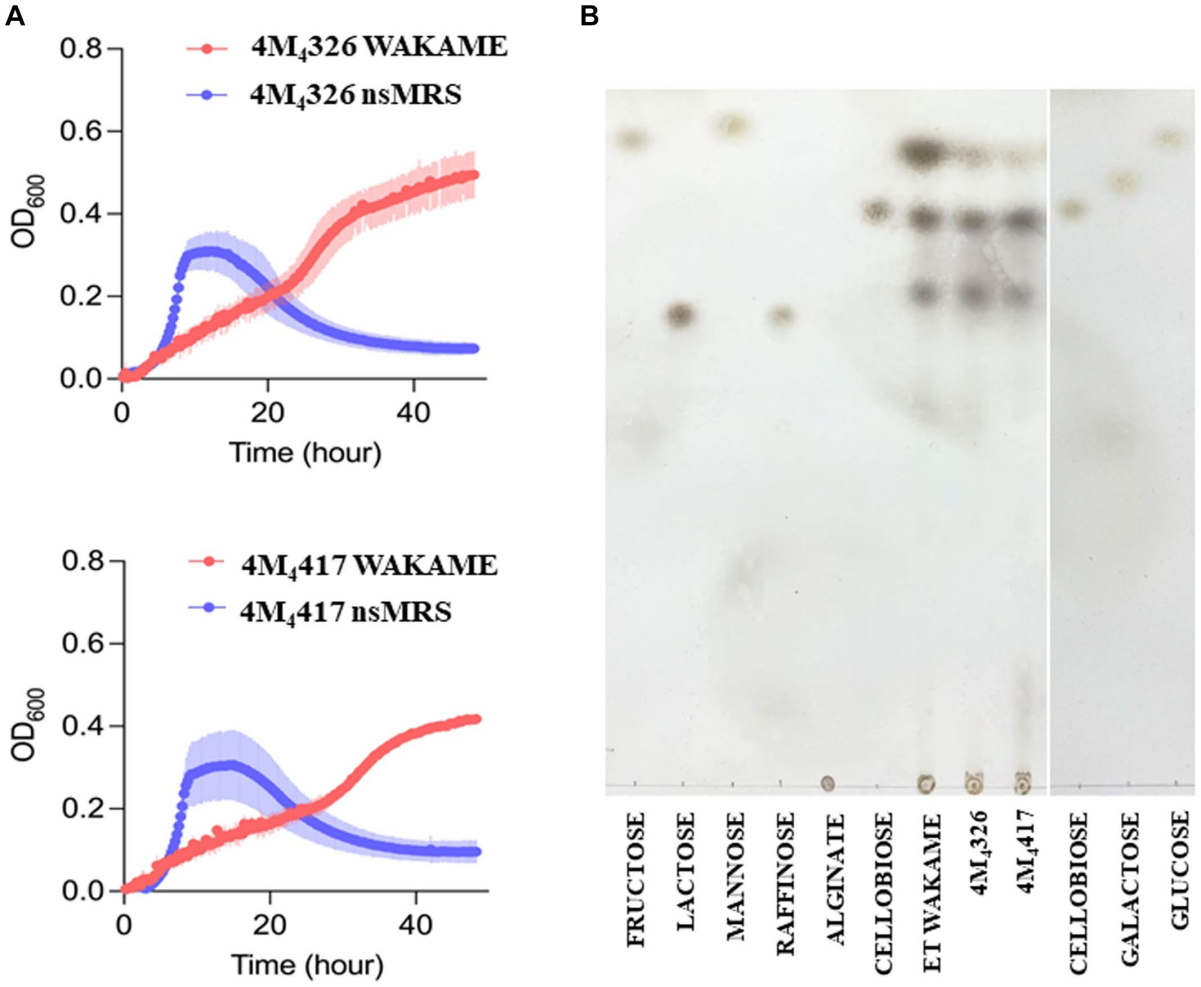
Growth of *Lactiplantibacillus plantarum* strains on wakame based-medium. The ability of 4M_4_326, and 4M_4_417 strains to utilize wakame to grow was evaluated estimating the optical density (OD) and compared with the growth in non-sugar MRS medium **(A)**. Thin layer chromatography of different sugar and wakame fermentation in nutrient broth **(B)**.

Wakame, an edible brown alga, contains various biologically active components that regulate the immune function (Jimenez-Escrig et al., 2011). Therefore, wakame has been proposed as a potential prebiotic feed supplement, however, only some microorganisms have the capacity to utilize its polysaccharides to grow (Tang et al., 2007). Our previous *in vivo* studies demonstrated that the administration of wakame to pigs as a feed significantly elevated the abundance of *Lactobacillus* spp. in the gut (Masumizu et al., 2019). Indeed, some *L. plantarum* strains isolated from the feces of wakame fed pig showed well growth in the presence of wakame *in vitro* (Masumizu et al., 2019). Therefore, wakame is expected to function as a natural prebiotic for selected *L. plantarum* strains. Accordingly, we conducted growth assay of *L. plantarum* 4M_4_326 and 4M_4_417 isolated in this work, on a non-sugar MRS medium supplemented with wakame as a sole carbon source (Figure 4A). In the early growth phase, both strains showed fast growth in the non-sugar MRS (control) medium, but the OD_600_ values decreased within 24 h. By contrast, in the wakame-supplemented medium, both strains exhibited a continuous growth pattern even after 40 h cultivation. TLC analysis indicated that the spots corresponding to monosaccharide were decreased in the culture medium of strains 4M_4_326 and 4M_4_417, compared to enzyme-treated wakame (Figure 4B). Of note, the Rf of the glucose standard did not match with the one from enzyme-treated wakame. Polysaccharide present in wakame seaweed is not completely degraded to pure monosaccharide like the standard glucose, and the glucose in the enzyme-treated wakame would be connected with some residues such as sulfates which are considered abundant in wakame. These results suggest that the wakame can enhance the growth of *L. plantarum* 4M_4_326 and 4M_4_417, although more studies are needed to determine the sugars present in the enzyme-treated wakame that favor the growth of lactobacilli. Remarkably there were other sugars in the TLC analysis that were not to be consumed by lactobacilli. The enzyme-treated wakame also contain disaccharides (cellobiose) and trisaccharide that cannot be assimilated by *L. plantarum* 4M4326 and 4M_4_417 but could be fermented by other intestinal bacteria in the porcine host when orally administered. Investigating the ability of the wakame fermented with 4M_4_326 or 4M_4_417 strains to modulate the composition of the porcine intestinal microbiota would be of value to completely characterize the potential beneficial effects of the symbiotic wakame-lactobacilli.

## Conclusion

In the present work, we demonstrated that sow milk can be an excellent source for the isolation of potential probiotic (immunobiotic) LAB strains. Our experiments indicated that two *L. plantarum* strains derived from pig milk have immunomodulatory activities in PIE cells since they are able to modulate the expressions of genes involved in innate immune responses. *L. plantarum* 4M_4_417 efficiently regulated the expression of PGRPs, pBD, and NODs indicating its potential to beneficially influence the innate defence mechanism of the porcine intestinal mucosa. On the other hand, *L. plantarum* 4M_4_326 improved the expression of type I IFNs in PIE cells indicating its potential to be used as a probiotic enhancer of antiviral immunity in the porcine gut. Further *in vivo* investigations are needed to document the immunomodulatory activities of the 4M_4_326 and 4M_4_417 strains in pigs. In addition, future *in vivo* research on the synergistic combination of the immunomodulatory effects of wakame and the *L. plantarum* strains selected here are necessary to advance in the development of a functional immunosymbiotic feed with the capacity to efficiently improve immune health status and reduce the severity of intestinal infections in weaned piglets.

## Data availability statement

All raw data from the microbiome analysis have been deposited in the following public database: DDBJ (https://www.ddbj.nig.ac.jp/index-e.html), accession no. PRJDB16792.

## Ethics statement

Ethical approval was not required for the studies on animals in accordance with the local legislation and institutional requirements because only commercially available established cell lines were used.

## Author contributions

RO: Formal analysis, Investigation, Methodology, Writing – original draft. SS: Formal analysis, Investigation, Methodology, Writing – original draft. BZ: Formal analysis, Investigation, Methodology, Writing – review & editing. TS: Formal analysis, Investigation, Methodology, Writing – review & editing. MS: Data curation, Formal analysis, Investigation, Methodology, Writing – review & editing. YN: Formal analysis, Investigation, Methodology, Writing – review & editing. FN: Data curation, Formal analysis, Investigation, Methodology, Writing – review & editing. WI-O: Data curation, Formal analysis, Investigation, Methodology, Writing – review & editing. YS: Data curation, Formal analysis, Investigation, Project administration, Resources, Writing – review & editing. KN: Data curation, Formal analysis, Investigation, Methodology, Resources, Writing – review & editing. JV: Funding acquisition, Investigation, Visualization, Writing – original draft. HK: Funding acquisition, Investigation, Project administration, Resources, Supervision, Writing – review & editing.

## Footnotes

^1^ https://doi.org/10.1038/s41587-019-0209-9
