# Supplementary material for: In vitro evaluation of the immunomodulatory and wakame assimilation properties of Lactiplantibacillus plantarum strains from swine milk
Source: Front Microbiol. 2024 Jan 26;15:1324999. doi: 10.3389/fmicb.2024.1324999 (PMC10858614; doi:10.3389/fmicb.2024.1324999)
Supplement: Supplementary file 1 [file Data_Sheet_1.docx]

**Table S1:** List of primers used in this study.

| Gene | Primer | Sequence (5'→3') | Reference |
| --- | --- | --- | --- |
| *β-Actin* | Forward | CAT CAC CAT CGG CAA CGA | Moue et al (2008) |
|  | Reverse | GCC CGC GAT GGT CTT G |  |
| *NOD1* | Forward | CTG TCG TCA ACA CCG ATC CA | Fukuyama et al. (2023) |
|  | Reverse | CCA GTT GGT GAC GCA GCT T |  |
| *NOD2* | Forward | GAC CGC ATC CTC TTA ACT TTC G | Fukuyama et al. (2023) |
|  | Reverse | ACG CTC GTG ATC CGT GAA C |  |
| *pBD1* | Forward | TGC CAC AGG TGC CGA TCT | Wang et al. (2019) |
|  | Reverse | CTG TTA GCT GCT TAA GGA ATA AAG |  |
| *pBD3* | Forward | CCT TCT CTT TGC CTT GCT CTT | Wang et al. (2019) |
|  | Reverse | GCC ACT CAC AGA ACA GCT ACC |  |
| *IFN-α* | Forward | CAG GGC AGA AGT CAT GAG ATC C | Tomokiyo et al. (2022) |
|  | Reverse | ATG AAC CAG GTG TCT GTC ACT C |  |
| *IFN- β* | Forward | AGT TGC CTG GGA CTC AA | Moue et al (2008) |
|  | Reverse | CCT CAG GGA CCT CAA AGT TCAT |  |

**Table S2**: List of isolated bacterial strains from sow milk

| Strain | Identified species | Identification (%) | Source | Day |
| --- | --- | --- | --- | --- |
| 4cs132 | *W. thailandensis* | 94 | Colostru m | day0 |
| 4cs143 | *Lc. lactis* | 91 | Colostru m | day0 |
| 4cs234 | *W. paramesenteroides* | 87 | Colostru m | day0 |
| 4cs236 | *W. cibaria* | 85 | Colostru m | day0 |
| 4cs32 | *W. param esenteroides* | 93 | Colostru m | day0 |
| 4cs321 | *L. plantarum* | 96 | Colostru m | day0 |
| 4cs322 | *W. thailandensis* | 91 | Colostru m | day0 |
| 4cs331 | *L. plantarum* | 95 | Colostru m | day0 |
| 4cs332 | *Lc. lactis* | 88 | Colostru m | day0 |
| 4cs335 | *Lc. lactis* | 91 | Colostru m | day0 |
| 4cs338 | *W. paramesenteroides* | 87 | Colostru m | day0 |
| 4cs342 | *Lc. lactis* | 91 | Colostru m | day0 |
| 4cs349 | *W. thailandensis* | 88 | Colostru m | day0 |
| 4cs423 | *Lc. lactis* | 98 | Colostru m | day0 |
| 4M_2_ 11 | *W. cibaria* | 92 | M ilk | day14 |
| 4M_2_ 12 | *W. cibaria* | 89 | M ilk | day14 |
| 4M_2_ 13 | *W. cibaria* | 92 | M ilk | day14 |
| 4M_2_ 16 | *W. cibaria* | 91 | M ilk | day14 |
| 4M_2_ 17 | *W. cibaria* | 93 | M ilk | day14 |
| 4M_2_ 18 | *W. cibaria* | 93 | M ilk | day14 |
| 4M_2_ 110 | *W. cibaria* | 92 | M ilk | day14 |
| 4M_2_ 111 | *W. cibaria* | 92 | M ilk | day14 |
| 4M_2_ 112 | *W. cibaria* | 91 | M ilk | day14 |
| 4M_2_ 113 | *W. cibaria* | 91 | M ilk | day14 |
| 4M_2_ 114 | *W. cibaria* | 91 | M ilk | day14 |
| 4M_2_ 115 | *W. cibaria* | 90 | M ilk | day14 |
| 4M_2_ 117 | *W. cibaria* | 93 | M ilk | day14 |
| 4M_2_ 118 | *W. cibaria* | 91 | M ilk | day14 |
| 4M_2_ 123 | *W. cibaria* | 91 | M ilk | day14 |
| 4M_2_ 125 | *W. cibaria* | 96 | M ilk | day14 |
| 4M_2_ 127 | *W. cibaria* | 92 | M ilk | day14 |
| 4M_2_ 128 | *W. cibaria* | 95 | M ilk | day14 |
| 4M_2_ 129 | *W. cibaria* | 91 | M ilk | day14 |
| 4M_2_ 130 | *W. cibaria* | 92 | M ilk | day14 |
| 4M_2_ 132 | *W. cibaria* | 91 | M ilk | day14 |
| 4M_2_ 133 | *W. cibaria* | 93 | M ilk | day14 |
| 4M_2_ 134 | *W. cibaria* | 93 | M ilk | day14 |
| 4M_3_ 58 | *Lc. garvieae* | 94 | M ilk | day21 |
| 4M_4_ 311 | *W. paramesenteroides* | 89 | M ilk | day28 |
| 4M_4_ 318 | *W. paramesenteroides* | 92 | M ilk | day28 |
| 4M_4_ 321 | *W. paramesenteroides* | 90 | M ilk | day28 |
| 4M_4_ 322 | *W. paramesenteroides* | 91 | M ilk | day28 |
| 4M_4_ 323 | *W. paramesenteroides* | 90 | M ilk | day28 |
| 4M_4_ 325 | *W. paramesenteroides* | 93 | M ilk | day28 |
| 4M_4_ 326 | *L. plantarum* | 96 | M ilk | day28 |
| 4M_4_ 327 | *W. paramesenteroides* | 93 | M ilk | day28 |
| 4M_4_ 328 | *W. paramesenteroides* | 93 | M ilk | day28 |
| 4M_4_ 329 | *W. paramesenteroides* | 93 | M ilk | day28 |
| 4M_4_ 330 | *W. paramesenteroides* | 92 | M ilk | day28 |
| 4M_4_ 331 | *W. paramesenteroides* | 90 | M ilk | day28 |
| 4M_4_ 333 | *W. paramesenteroides* | 93 | M ilk | day28 |
| 4M_4_ 334 | *W. paramesenteroides* | 90 | M ilk | day28 |
| 4M_4_ 335 | *W. paramesenteroides* | 89 | M ilk | day28 |
| 4M_4_ 336 | *W. paramesenteroides* | 88 | M ilk | day28 |
| 4M_4_ 337 | *W. paramesenteroides* | 94 | M ilk | day28 |
| 4M_4_ 338 | *L. plantarum* | 96 | M ilk | day28 |
| 4M_4_ 339 | *W. paramesenteroides* | 87 | M ilk | day28 |
| 4M_4_ 340 | *W. paramesenteroides* | 92 | M ilk | day28 |
| 4M_4_ 341 | *W. paramesenteroides* | 94 | M ilk | day28 |
| 4m_4_ 342 | *W. paramesenteroides* | 84 | M ilk | day28 |
| 4M_4_ 346 | *L. plantarum* | 94 | M ilk | day28 |
| 4M_4_ 347 | *L. plantarum* | 97 | M ilk | day28 |
| 4M_4_ 348 | *W. paramesenteroides* | 92 | M ilk | day28 |
| 4M_4_ 349 | *W. paramesenteroides* | 92 | M ilk | day28 |
| 4M_4_ 350 | *W. paramesenteroides* | 91 | M ilk | day28 |
| 4M_4_ 352 | *W. paramesenteroides* | 93 | M ilk | day28 |
| 4M_4_ 353 | *W. paramesenteroides* | 92 | M ilk | day28 |
| 4M_4_ 354 | *W. paramesenteroides* | 90 | M ilk | day28 |
| 4M_4_ 355 | *W. paramesenteroides* | 92 | M ilk | day28 |
| 4M_4_ 356 | *W. paramesenteroides* | 92 | M ilk | day28 |
| 4M_4_ 357 | *W. paramesenteroides* | 94 | M ilk | day28 |
| 4M_4_ 358 | *W. paramesenteroides* | 92 | M ilk | day28 |
| 4M_4_ 359 | *W. paramesenteroides* | 90 | M ilk | day28 |
| 4M_4_ 360 | *W. paramesenteroides* | 92 | M ilk | day28 |
| 4M_4_ 361 | *W. paramesenteroides* | 91 | M ilk | day28 |
| 4M_4_ 362 | *W. paramesenteroides* | 93 | M ilk | day28 |
| 4M_4_ 363 | *W. paramesenteroides* | 91 | M ilk | day28 |
| 4M_4_ 364 | *W. paramesenteroides* | 90 | M ilk | day28 |
| 4M_4_ 365 | *W. paramesenteroides* | 95 | M ilk | day28 |
| 4M_4_ 366 | *W. paramesenteroides* | 90 | M ilk | day28 |
| 4M_4_ 367 | *W. paramesenteroides* | 93 | M ilk | day28 |
| 4M_4_ 368 | *W. paramesenteroides* | 94 | M ilk | day28 |
| 4M_4_ 369 | *W. paramesenteroides* | 91 | M ilk | day28 |
| 4M_4_ 37 | *W. paramesenteroides* | 92 | M ilk | day28 |
| 4M_4_ 39 | *Lc. lactis* | 90 | M ilk | day28 |
| 4M_4_ 51 | *W. paramesenteroides* | 91 | M ilk | day28 |
| 4M_4_ 53 | *Lc. lactis* | 88 | M ilk | day28 |
| 4M_4_ 54 | *Lc. lactis* | 90 | M ilk | day28 |
| 4M_4_ 370 | *W. paramesenteroides* | 96 | M ilk | day28 |
| 4M_4_ 372 | *W. paramesenteroides* | 89 | M ilk | day28 |
| 4M_4_ 373 | *W. cibaria* | 92 | M ilk | day28 |
| 4M_4_ 374 | *W. paramesenteroides* | 93 | M ilk | day28 |
| 4M_4_ 412 | *W. paramesenteroides* | 89 | M ilk | day28 |
| 4M_4_ 417 | *L. plantarum* | 94 | M ilk | day28 |
| 4M_4_ 419 | *W. paramesenteroides* | 89 | M ilk | day28 |
| 4M_4_ 428 | *W. paramesenteroides* | 92 | M ilk | day28 |
| 4M_4_ 433 | *W. paramesenteroides* | 91 | M ilk | day28 |
| 4M_4_ 435 | *W. paramesenteroides* | 88 | M ilk | day28 |
| 4M_4_ 437 | *W. paramesenteroides* | 92 | M ilk | day28 |
| 4M_4_ 442 | *W. paramesenteroides* | 89 | M ilk | day28 |
| 4M_4_ 444 | *W. paramesenteroides* | 92 | M ilk | day28 |
| 4M_4_ 448 | *Lc. lactis* | 93 | M ilk | day28 |
| 4M_4_ 449 | *Lc. lactis* | 87 | M ilk | day28 |
| 4M_4_ 450 | *Lc. lactis* | 95 | M ilk | day28 |
| 4M_4_ 455 | *W. cibaria* | 97 | M ilk | day28 |
| 4M_4_ 456 | *W. cibaria* | 96 | M ilk | day28 |
| 4M_4_ 457 | *W. cibaria* | 93 | M ilk | day28 |
| 4M_4_ 459 | *W. cibaria* | 94 | M ilk | day28 |
| 4M_4_ 461 | *W. cibaria* | 98 | M ilk | day28 |
| 4M_4_ 462 | *W. cibaria* | 95 | M ilk | day28 |
| 4M_4_ 511 | *W. paramesenteroides* | 92 | M ilk | day28 |
| 4M_4_ 517 | *W. paramesenteroides* | 92 | M ilk | day28 |
| 4M_4_ 518 | *Lc. lactis* | 91 | M ilk | day28 |
| 4M_4_ 523 | *Lc. lactis* | 95 | M ilk | day28 |
| 4M_4_ 526 | *Lc. lactis* | 91 | M ilk | day28 |
| 4M_4_ 527 | *Lc. lactis* | 86 | M ilk | day28 |
| 4M_4_ 528 | *Lc. lactis* | 87 | M ilk | day28 |
| 4M_4_ 532 | *W. paramesenteroides* | 95 | M ilk | day28 |
| 4M_4_ 535 | *Lc. lactis* | 93 | M ilk | day28 |
| 4M_4_ 537 | *Lc. lactis* | 93 | M ilk | day28 |
| 4M_4_ 538 | *W. paramesenteroides* | 92 | M ilk | day28 |
| 4M_4_ 539 | *Lc. lactis* | 94 | M ilk | day28 |
| 4M_4_ 541 | *W. paramesenteroides* | 91 | M ilk | day28 |
| 4M_4_ 542 | *W. paramesenteroides* | 93 | M ilk | day28 |
| 4M_4_ 546 | *W. paramesenteroides* | 90 | M ilk | day28 |
| 4M_4_ 547 | *W. paramesenteroides* | 94 | M ilk | day28 |
